# How pain management for children with cerebral palsy in South African schools complies with up-to-date knowledge

**DOI:** 10.4102/ajod.v8i0.575

**Published:** 2019-11-22

**Authors:** Ensa Johnson, Stefan Nilsson, Margareta Adolfsson

**Affiliations:** 1 Centre for Augmentative and Alternative Communication, Faculty of Humanities, University of Pretoria, Pretoria, South Africa; 2 Children, Health, Intervention, Learning and Development (CHILD), Jönköping University, Jönköping, Sweden; 3 Institute of Health and Care Sciences, University of Gothenburg, Gothenburg, Sweden; 4 School of Education and Communication, Jönköping University, Jönköping, Sweden; 5 Swedish Institute of Disability Research, Jönköping University, Jönköping, Sweden

**Keywords:** evidence-based practice, intervention, clinicians, children with cerebral palsy, pain management

## Abstract

**Background:**

Pain in children with cerebral palsy (CP) has its sources in musculoskeletal problems that can influence learning in a school setting. Best pain management is essential for these children, but school staff may not keep up to date with the latest developments and interventions. Therefore, staff’s perceptions of beneficial strategies may not comply with contemporary scientific knowledge about effective evidence-based interventions.

**Objectives:**

This study investigated how pain management intervention for children with CP in South African schools complied with international scientific knowledge about evidence-based interventions. The intention was to provide support for an update of knowledge on both individual level (i.e. professionals) and system level (i.e. decision makers).

**Method:**

Five focus groups were conducted with staff members at five schools for children with special educational needs in South Africa. Manifest and latent content analyses of professional statements identified interventions reported as beneficial and related them to higher and lower levels of intervention evidence as reported at the time of data collection.

**Results:**

Most treatment strategies concerned motor functioning that fell within the framework of physiotherapists and occupational therapists. Access to orthopaedic expertise was limited, waiting times were long and medication for spasticity treatment was not offered.

**Conclusion:**

A discrepancy between published evidence and clinical practice for pain management in children with CP in South African school settings was noted. Suggestions for improved early intervention to identify children’s hips at risk through surveillance programmes; and orthopaedic management are proposed to prevent deformities and unnecessary suffering in South African children with CP.

## Introduction

For children with disability, it is essential to provide current and evidence-based interventions to ensure best treatment. Professionals may find it difficult to keep up to date with the latest developments in cerebral palsy (CP) intervention because of the substantial increase of systematic reviews published on CP intervention in the last decade (Anttila et al. 2008; Donald et al. 2014; Novak et al. 2013; Reedman, Boyd & Sakzewski 2017; Thomason & Graham 2014; Wiart, Darrah & Kembhavi 2008). Novak and her Australian colleagues reported on the state of evidence for interventions to specifically support children with CP (Novak 2014; Novak et al. 2013). Even though the results of Novak’s comprehensive systematic review were questioned by some Australian experts as these experts were of the opinion that the majority of the studies included in the review seemed to be sponsored by industry (Thomason & Graham 2014), it is important to consider effective evidence-based CP interventions apart from surgery. In school settings, pain management could contribute to children with CP’s optimal learning opportunities and participation in classroom activities. This study, which is part of a larger project that aims to facilitate discussions about best practices (Adolfsson, Johnson & Nilsson 2018; Johnson, Nilsson & Adolfsson 2015; Nilsson, Johnson & Adolfsson 2016), explores how professionals working in South African schools for children with special educational needs perceive pain management intervention for children with CP, and how their ideas about interventions comply with knowledge about evidence-based interventions as reported by Novak et al. (2013).

## Pain in cerebral palsy

Cerebral palsy includes a group of permanent disorders of the development of movement and posture (Rosenbaum, Paneth & Leviton 2007) that could limit persons with CP to participate in activities of daily living (such as eating, sleeping and walking), physical activities (such as gross and fine motor activities), communication, learning and social engagement (Breau 2011; Johnson et al. 2015; Lauruschkus et al. 2017). In addition to individual factors, environmental factors may further impact the independence of a child living with CP, such as physical accessibility, lack of assistive materials or support networks (Novak et al. 2013).

The focus of this article will be on the classification of chronic nociceptive musculoskeletal pain according to the International Classification of Diseases 11th Revision (ICD-11; World Health Organization 2019). Chronic nociceptive musculoskeletal pain is often under-recognised even though it is the most common comorbidity in children with CP (Novak 2014; Westbom, Rimstedt & Nordmark 2017). Causes of pain may be multi-factorial and could be attributed to spasticity (occurring in 75% of children with CP), contractures (in 80% of children with CP) and/or inability to walk (in 33% of children with CP) (Novak 2014). Children with spasticity are largely at risk of contractures. Contractures may lead to hip displacement and further progress into painful hip dislocation. Hip dislocation means that the femoral head is completely displaced laterally out of the acetabulum (Hägglund et al. 2016). Displacement and/or dislocation of the hip is a major hindrance for children with CP. It is present in one out of three children with CP, manifesting itself from 2 to 3 years of age (Huser, Mo & Hosseinzadeh 2018; Novak et al. 2013). Children with bilateral involvement who cannot walk have the highest risk of hip displacement or dislocation. In severe cases, a windswept syndrome may occur, sometimes combined with scoliosis (Hägglund et al. 2016). The windswept hip deformity usually starts from the lower extremities with abduction and external rotation of one hip, with the opposite hip in adduction and internal rotation (Hägglund et al. 2016). Windswept syndrome is difficult to treat and could affect a child’s ability to stand or make sitting and any lying down uncomfortable.

## Pain management

Research in recent years has proven that prevention approaches could manage a child’s pain and other comorbidities, such as hip displacement, epilepsy or sleep disorders, as well as lessen the development of contractures that could worsen the child’s health outcome (Donald et al. 2014; Hägglund et al. 2014). One means of prevention is to systematically follow-up the health status of children with CP, with a focus on the hip displacement and muscle contractures. Systematic hip surveillances have been created by orthopaedic surgeons in collaboration with therapists and used successfully in, for example, Nordic countries, Scotland and Australia (Hägglund et al. 2014; Wynter et al. 2014). However, no such systematic management process or preventive pharmacological treatment is available in South Africa (Donald et al. 2014; Nilsson et al. 2016).

## Intervention strategies in South African school settings

In recent studies, professionals (teachers and therapists) working with children with CP in school settings in South Africa and Sweden were asked about the intervention strategies they used to manage the children’s recurrent pain in school settings (Adolfsson et al. 2018; Johnson et al. 2015; Nilsson et al. 2016). The professionals in the said studies reported on a variety of intervention strategies. Subsequently, action-and-reaction approaches followed by healthcare professionals during pain management were identified (Adolfsson et al. 2018). Action refers to strategies professionals use to prevent pain, while reaction refers to professionals’ strategies to intervene when a child is already experiencing pain. A difference was noted between strategies employed by South African and Swedish professionals, as a reaction approach was more commonly used in South Africa (Adolfsson et al. 2018). Reasons for this finding could be that the Swedish health professionals (who are typically consultants in school settings) had access to interventions to prevent children’s pain. Such interventions are a systematic follow-up surveillance programme for children with CP – also referred to as the Cerebral Palsy follow-Up Programme (CPUP) (Degerstedt, Wiklund & Enberg 2017), as well as pharmacological pain management strategies, for example, botulinum toxin (BoNT) and baclofen intended for the reduction of spasticity and recurrent pain (Hägglund et al. 2014). In contrast, in South Africa, pain management strategies are based on traditional analgesics, that is, paracetamol and non-steroidal anti-inflammatory agents. These drugs are mostly used for acute pain (Wren et al. 2019). In the South African schools where the study was conducted, teachers or nurses sometimes only offered children a glass of water as an alternative to pain medication (Adolfsson et al. 2018; Nilsson et al. 2016). Possible explanations for strategies used by South Africa professionals working in school settings are (1) legislation stipulating that only doctors can prescribe pharmacological treatment, (2) caregivers (e.g. participants in the said study) avoiding analgesics because of potential side effects, (3) medical doctors and orthopaedic surgeons who are only available outside school settings and (4) patients (e.g. children with CP) who have to wait very long periods to get appointments with specialists in public hospitals (Nilsson et al. 2016).

In comparison with Swedish healthcare professionals, the school-based South African health professionals were able to provide ‘hands-on’ or immediate pain management when a child experienced pain. These action–reaction approach differences may also be the reason why there is a higher incidence of children with severely affected CP (Gross Motor Function Classification System [GMFCS] levels IV and V) reported in Africa compared to those in European countries (such as Sweden) and North America (Donald et al. 2014, 2015). Nevertheless, one previous study in this project (Adolfsson et al. 2018) found that the South African participants valued other action interventions implemented globally to support the well-being of children with CP. They suggested additional resources needed to secure a satisfactory pain management intervention for these children.

## State of the evidence of interventions for children with cerebral palsy

A comprehensive systematic meta-review was conducted by Novak et al. (2013) to obtain current knowledge about the best available published intervention evidence for children with CP. Data in the current study have been sorted and analysed based on the results and recommendations by Novak (2014) and Novak et al. (2013).

### Interventions by outcome

Novak et al. (2013) reported intervention options for 10 categories of outcomes. Only three of the categories that focused on pain management were considered relevant for the current study (1) spasticity management, (2) contracture management and (3) improved motor activities and function (see Appendix 1). The other seven categories mentioned by Novak et al. (2013) that were not within the scope of this manuscript included mealtime management, improved muscle strength, self-care, communication, behaviour and social skills, parent coping and bone density.

### Evidence of interventions

Evidence of a variety of interventions was reported in the meta-review by Novak et al. (2013). However, all intervention options did not necessarily focus on pain management. For example, constraint-induced movement therapy (CIMT), bimanual training and occupational training following BoNT aim to improve functioning in the upper limbs rather than to reduce pain. The effectiveness of interventions was based on the framework of Grading of Recommendations Assessment, Development and Evaluation (GRADE). Grading of Recommendations Assessment, Development and Evaluation is developed to assess efficiency and formulate recommendations along a four-part continuum:

- S+. Strong evidence for (‘Do it’)
- W+. Conditional for (‘Probably do it’)
- W-. Conditional against (‘Probably don’t do it’)
- S-. Strong evidence against (‘Don’t do it’).

Table 1 explains the levels of evidence for intervention options relevant for pain management related to the three selected categories of outcomes from Novak et al. (2013). For a more detailed explanation of these interventions, refer to Appendix 1.

**TABLE 1:** Levels of evidence for a sample of pain management intervention options related to the three categories of expected outcomes from Novak et al. (2013).

Level of evidence	Intervention options/outcome		
	Spasticity management†	Contracture management†	Improved motor activities and function†
S+ ‘Do it’	Botulinum toxin (BoNT) Diazepam Selective dorsal rhizotomy	Casting lower limb‡	Bimanual training Constraint-induced movement therapy (CIMT) Context-focused therapy Goal-directed functional training Occupational training following BoNT home programmes (parent training included)‡
W+ ‘Probably do it’	Baclofen oral Intrathecal baclofen (ITB) Tizanidine oral	Ankle foot orthotics (AFOs) Casting upper limb‡ Orthopaedic interventions (hip and other surgery) Orthotic hand Hand surgery Single-event multilevel surgery (SEMLS)	Acupuncture assistive technology‡ Biofeedback Early intervention‡ Electrical stimulation‡ Hippotherapy‡ Hydrotherapy‡ Seating and positioning (including pressure care)‡ SEMLS and therapy‡
W- ‘Probably don’t do it’	Casting (upper and lower limbs) Dantrolene Intramuscular injections of alcohol or phenol	Stretching manual‡	Conductive education TheraSuits§ Vojta¶
S-	Hip bracing	NDT‡	NDT‡
‘Don’t do it’	-	-	Hyperbaric oxygen Sensory integration (SI)

*Source*: Novak, I., McIntyre, S., Morgan, C., Campbell, L., Dark, L., Morton, N. et al., 2013, ‘A systematic review of interventions for children with cerebral palsy: State of the evidence’, *Developmental Medicine & Child Neurology* 55(10), 885–910. https://doi.org/10.1111/dmcn.12246

NDT, neurodevelopmental therapy.

† , Intervention options determined as most relevant for pain management.

‡ , Interventions reported by participants as beneficial for the child’s pain management.

§ , Exercise technique using a soft dynamic proprioceptive orthotic device to facilitate motor performances of children with CP (Alagesan & Shetty 2010).

¶ , A ‘dynamic neuromuscular treatment method based on the developmental kinesiology and principles of reflex locomotion’ (Vojta 2019).

From Table 1, it is clear that interventions such as neurodevelopmental therapy (NDT), casting or hip bracing to reduce spasticity do not have sufficient levels of evidence (W- and S-) to prove them to be beneficial interventions to treat pain in children with CP (Novak et al. 2013) as some healthcare professionals tend to believe (Nilsson et al. 2016). It is further important to mention that according to Novak et al.’s (2013) level of evidence, lower limb casting has S+ evidence for contracture management but W- for spasticity management – it is this kind of discrepancy that makes it difficult for healthcare professionals to discern treatment choices. Therefore, it is important for researchers and healthcare professionals to determine if the current intervention practices used are evidence based and could, in fact, support pain management in children with CP.

## Aim

This study focused on pain management for children with CP in South African schools for children with special educational needs. The aim was to investigate how interventions that the professionals reported as beneficial for the child’s pain management complied with published international evidence-based interventions for children with CP, as reported by Novak et al. (2013) at the time of data collection. Another aim was to reflect on the participants’ perceptions about interventions or resources perceived missing to obtain and secure a satisfactory pain management strategy for children with CP. The intention was to provide support for an update of knowledge on both individual level (i.e. professionals) and system level (i.e. decision makers).

## Research methods and design

The study followed a descriptive with a directed, qualitative approach (Hsieh & Shannon 2005). Using a combination of manifest and latent content analyses (Graneheim & Lundman 2004), professional statements from focus groups were analysed and reflected against published interventions for CP reported as effective by Novak et al. (2013, 2014). The study is limited to pain management in children with CP even though the larger project, of which this study is a part, addressed pain assessment and pain management (Adolfsson et al. 2018; Johnson et al. 2015; Nilsson et al. 2016). Ethics approval was obtained from the Research Ethics Committee of the University (GW20140201HS) and permission was granted from the Gauteng Department of Education (D2014/226), South Africa.

## Setting

Principals from five public schools for children with special educational needs which accommodate children with CP in the Gauteng area of South Africa gave permission for focus groups to be conducted with staff at their schools in February 2014. The school principals (or a designated person appointed by the principal) completed a short questionnaire to provide the investigators with detailed information on the school, for example, the ages of the children accommodated in the school, the number of children in the school, the number of children with CP in the school as well as whether the school has a hostel facility (see Table 2). The participating schools accommodated children with various types of disabilities, including CP. Three of the five schools were boarding schools where children from rural areas were accommodated in hostels. As the children from rural areas had to attend a boarding school, they typically start schooling at the age of seven. It was reported by some of the participants that these children received limited to no early intervention (EI) services, resulting in hip displacement or dislocations and chronic nociceptive musculoskeletal pain already present.

**TABLE 2:** Background information of schools included in the study.

School information	School A	School B	School C	School D	School E
Ages of children (years)	13–21	3–21	6–18	3–21	3–21
Number of children	506	403	329	372	270
Number of children with CP	16	106	126	241	100
Hostel (boarding facilities)	Yes (100%)	Yes (6%)	No	Yes (27%)	No
Number of teachers	53	49	28	34	20
Number of clinicians	7	21	6	2	8

*Source*: Johnson, E., Nilsson, S. & Adolfsson, M., 2015, ‘Eina! Ouch! Eish! Professionals’ perceptions of how children with cerebral palsy communicate about pain in South African school settings: Implications for the use of AAC’, *Augmentative and Alternative Communication* 31(4), 325–335. https://doi.org/10.3109/07434618.2015.1084042; Nilsson, S., Johnson, E. & Adolfsson, M., 2016, ‘Professionals perceptions about the need for pain management interventions for children with cerebral palsy in South African school settings’, *Pain Management Nursing* 17(4), 249–226. https://doi.org/10.1016/j.pmn.2016.03.002

CP, cerebral palsy.

The number of children at the five schools varied from 270 to 506, of whom 16–241 (3% – 65%) were diagnosed with CP (Table 2).

## Participants

Upon consent, 38 staff members from the five schools participated in five separate focus groups. Criteria for inclusion were follows: any staff member (e.g. teachers, therapists, psychologists, social workers and personal assistants) who worked at the five schools with children with CP (see Table 3). All participants were women except for one who was the sibling and personal assistant of a child with severe CP. The age range of the participants was 22–64 years, with an average age of 44.4 years. In total, 76% of the 11 teacher participants had at least 4 years of university education with an average of 11.8 years experience in working with children with CP, attesting to their knowledge of this condition. The 26 participating clinicians represented six professions: nursing (*n* = 5), occupational therapy (OT; *n* = 8), physiotherapy (PT; *n* = 6), psychology (*n* = 1), social work (*n* = 1) and speech-language therapy (*n* = 5). Therapists worked in therapy rooms and school classrooms with the children.

**TABLE 3:** Background information of the focus group participants at each school.

Focus group (FG) participants	FG 1	FG 2	FG 3	FG 4	FG 5
Teacher	1	2	1	2	3
Special needs teacher	1	0	1	0	0
Nurse	0	1	1	2	1
Occupational therapist	1	1	1	0	5
Physiotherapist	2	1	1	1	1
Psychologist	0	1	0	0	0
Social worker	0	0	1	0	0
Speech and language therapist	1	2	1	1	0
Personal assistant	0	0	0	1	0

*Source*: Adolfsson, M., Johnson, E. & Nilsson, S., 2018, ‘Pain management for children with cerebral palsy in school settings in two cultures: action and reaction approaches’, *Disability and Rehabilitation* 40(18), 2152–2162. https://doi.org/10.1080/09638288.2017.1327987; Nilsson, S., Johnson, E. & Adolfsson, M., 2016, ‘Professionals perceptions about the need for pain management interventions for children with cerebral palsy in South African school settings’, *Pain Management Nursing* 17(4), 249–226. https://doi.org/10.1016/j.pmn.2016.03.002

## Data collection

The five focus group interview sessions included two identically applied parts, focusing on pain assessment and pain management, respectively. In the beginning of each part, one main question was presented, followed by three supporting sub-questions (Table 4). Piloted interview guides in English directed the introduction of the topic and the performance of the focus groups. Three researchers, of whom two were Swedish and one South African, conducted the focus group interviews that lasted between 70 and 110 min per session. The third investigator (a physiotherapist [PT] having experience with children with CP as well as conducting focus groups) acted as the moderator (Wibeck et al. 2007). The first investigator (a special needs teacher who specialises in pain communication) typed all statements on a laptop and assisted the participants in Afrikaans where necessary. The second investigator (a paediatric nurse with specialisation in pain management) reflected on the statements and asked for more information when needed. The statements were projected onto a wall as they were typed, and the discussions were audio-recorded to be used as a reference for the researchers’ data analysis. At the end of the sessions, a member check was performed. All statements were jointly reviewed and revised or extended where necessary. To validate the data and enable participants to determine that the statements truly represented their experiences, two final questions were asked (White & Verhoef 2005): (1) do these findings accurately represent your experiences? and (2) is there anything we have missed that you feel should be included? For the purpose of this study, statements considered included professionals’ information about their use of methods, knowledge about methods that would be beneficial in their opinion and information about interventions or resources they perceived missing to address pain management of children with CP.

**TABLE 4:** Questions and supporting sub-questions used during focus group interviews.

Focus	Main question	Sub-question
Pain assessment	1. What are your experiences of persistent pain in children with CP in your school?	1.1. How do you observe a child with CP in pain? 1.2. How do you communicate pain to the child with CP? 1.3. How do children with CP communicate their pain?
Pain management	2. Which strategies and actions for pain management do you use to try to support children with CP to become active participants in the school activities, despite acute and chronic pain?	2.1. How would you act when a child with CP is in pain? 2.2. Which other strategies have you tried to manage pain in children with CP? 2.3. If you could do anything, what would you like to do?

*Source*: Adolfsson, M., Johnson, E. & Nilsson, S., 2018, ‘Pain management for children with cerebral palsy in school settings in two cultures: action and reaction approaches’, *Disability and Rehabilitation* 40(18), 2152–2162. https://doi.org/10.1080/09638288.2017.1327987; Johnson, E., Nilsson, S. & Adolfsson, M., 2015, ‘Eina! Ouch! Eish! Professionals’ perceptions of how children with cerebral palsy communicate about pain in South African school settings: Implications for the use of AAC’, *Augmentative and Alternative Communication* 31(4), 325–335. https://doi.org/10.3109/07434618.2015.1084042; Nilsson, S., Johnson, E. & Adolfsson, M., 2016, ‘Professionals perceptions about the need for pain management interventions for children with cerebral palsy in South African school settings’, *Pain Management Nursing* 17(4), 249–226. https://doi.org/10.1016/j.pmn.2016.03.002

CP, cerebral palsy.

## Data analysis

The statements (mostly equal to one meaning unit) were entered on separate spreadsheets for the five focus groups. A few comprehensive statements included two meaning units (Graneheim & Lundman 2004). For analyses in this study, all statements related to pain management were merged onto one spreadsheet, that is, both statements related to current strategies and requested additional resources. Reductions were made because of duplications and statements not related to CP or pain experiences.

All statements were initially reviewed and interpreted together by all investigators. Because the strategies about pain management often included several underlying meanings, the statements had to be interpreted according to the context, which is why a manifest approach was not sufficient. Statements about interventions that focused directly on pain management were linked to one of the categories of evidence-based intervention options for children with CP, as identified by Novak et al. (2013), and for the purpose of this study determined as most relevant, that is, spasticity management, contracture management or improved motor activities and function.

In the next step, the statements were condensed, labelled with a code and sorted into two categories that had previously been identified in the larger project by Nilsson et al. (2016): treatment strategies (i.e. ‘hands-on’ or immediate treatment strategies and medication to relieve pain) and environmental strategies (i.e. interventions influencing a child’s environment). To be clear about the underlying latent content in statements, all three investigators reviewed the linkages and jointly discussed professional interpretations. Thereby, the interpretation could lean on a multidisciplinary background knowledge. Finally, identified interventions were analysed and related to the updated knowledge reported in the contemporary meta-review by Novak et al. (2013). Before the discussion of results, the four levels of evidence were dichotomised into higher and lower levels to indicate intervention options that should be used (S+ and W+) and that should not be used (W- and S-) for pain management.

### Ethical considerations

Ethics approval was obtained from the Research Ethics Committee of the Faculty of Humanities at the University of Pretoria (GW20140201HS) and permission from the Gauteng Department of Education (D2014/226) in South Africa.

## Results and discussion

The five focus groups generated 164 statements that included information about available intervention options and 47 statements on the perceived need for additional interventions or resources. For the analysis, 21 of the statements about additional resources were excluded as they did not focus on pain management but rather on the need for extended resources. Examples of such resources were emotional support and speech training, help with self-care and schoolwork, or support at home that were not the focus of this article.

From one of the statements, one could understand how a child in pain might affect everyone around:

Keeping mind off the pain – she will smile and relax (gets quite tense when she is in pain) – peers in classroom also struggle to attend to their work – they feel sorry for them. (Teacher in FG4, Statement 49)

Another statement indicated the breadth of the problems that could exist for the children with CP and that could be taken into consideration in a school setting:

Constipation – put them on a mat or standing frame helps with constipation – work in a standing frame they are active participants, tables on different levels (basic standing frame). (PT in FG5, Statement 12)

These comments showed that not only musculoskeletal problems but also gastrointestinal dysfunction may cause pain in children with CP (Engel & Kartin 2006). Novak et al. (2012) also reported that constipation is a problem in more than 25% of children with CP.

Findings showed that the participants’ ideas about strategies beneficial for pain management of children with CP could fit into the three categories of outcomes as identified by Novak et al. (2013) and relevant for pain management (see Table 1). The subsequent discussion of the results will thus reflect participants’ concerns of their compliance with up-to-date knowledge as reported by Novak et al. (2013) (Table 5).

**TABLE 5:** Treatment and environmental strategies as reported by participants as beneficial for children’s pain management based on Novak et al.’s (2013) levels of the evidence of interventions for children with cerebral palsy.

Level of evidence	Treatment strategies	Environmental strategies
Higher levels of evidence: S+ ‘Do it’ W+ ‘Probably do it’	Home programmes Pressure care Seating and positioning Early intervention Parent training Hydrotherapy Electrical stimulation Hippotherapy	Casting lower limb
Lower levels of evidence: W- ‘Probably don’t do it’ S- ‘Don’t do it’	Stretching manual Neurodevelopmental therapy (NDT)	-

*Source*: Novak, I., McIntyre, S., Morgan, C., Campbell, L., Dark, L., Morton, N. et al., 2013, ‘A systematic review of interventions for children with cerebral palsy: State of the evidence’, *Developmental Medicine & Child Neurology* 55(10), 885–910. https://doi.org/10.1111/dmcn.12246

## Current intervention options

### Treatment strategies with higher-level evidence

Most of the treatment strategies discussed during the focus groups concerned motor activities and functioning (see Table 1). Not all statements really explained what it was about, but typically physiotherapy plays a central role in managing CP (Anttila et al. 2008). Motor activities focus on gross motor functioning and mobility and PTs use a variety of physical approaches to promote the well-being of a child. The role of a PT is related to that of an occupational therapist, who mostly focuses on fine motor functioning. Both of these professions teach caregivers how to handle their child at home and recommend mobility devices when needed. Many interventions mentioned by the participants fell within the framework of these two professions, and teachers seemed to lean against them as they often referred a child in pain to a therapist or asked the therapist to come to the classroom to support the child.

*Early intervention*, that is, child rehabilitation, and orthopaedic management must be prioritised because even if it cannot lessen the severity of the condition, it can stop the worsening of the status of CP and improve the child’s well-being (Herskind, Greisen & Nielsen 2015; Novak et al. 2017). Because of musculoskeletal problems, a child with CP can become progressively worse without intervention that could lead to nociceptive musculoskeletal pain. Therefore, early special care should be provided to children with CP in South Africa as CP cannot be cured. In addition, EI may have the potential to prevent chronic nociceptive musculoskeletal pain arising from non-treatment at an early age. To become effective, Novak (2014) reports that EI should be child-active, repetitive and structured, including gross and fine motor functions. Even though the meta-review by Novak et al. (2013) does not specifically focus on pain-related interventions, it is well known that pain in children with CP has its sources in hip dislocation, scoliosis, spasticity, tension, short muscles (i.e. contractures) and posture (Novak 2014; Stähle-Öberg & Fjellman-Wiklund 2009). Therefore, one can assume that improved mobility affects pain favourably. For example, because the child can load the skeleton to reduce spasticity, perform movements that prevent contractures, and adjust the position in sitting and standing.

It is further suggested that goal-directed functional training to improve motor activities should be included in the standard care of children with CP (Novak et al. 2013) to prevent chronic nociceptive musculoskeletal pain. The goals are set together with the family to make them meaningful and realistic, while the performance of interventions is simultaneously discussed. Tasks that are considered important enhance the child’s motivation and lead to more frequent training, especially if they are experienced as fun and mean improved participation in desired contexts (Novak 2014; Rosenbaum & Gorte 2012). Three specific intervention options with higher level of evidence were mentioned by the participants: hydrotherapy, electrical stimulation and hippotherapy. However, it was not obvious to what extent they were available.

Context-focused therapy is another compensatory approach that could be used from early age. It is compensatory but not focused on the child. Instead, the task or the environment is changed to promote the child’s successful task performance. In school settings, training could be integrated during classes by using standing positions, arranging materials to force reaching movements or short walks (Adolfsson et al. 2018).

*Seating and positioning* (including pressure care) were frequently mentioned by both teachers and therapists who seemed aware of its importance. In addition to preventing or reducing pain, correct seating and positioning could prevent contractures or scoliosis (Novak et al. 2017), improve hand and arm functioning (Cans, De-la-Cruz & Mermet 2008) or reduce the risk of pressure ulcers. It was a positive fact that all participants seemed to understand the importance of this intervention option.

*Home programmes and parent training* generally aim to improve the motor activity performance of children with CP (Novak et al. 2013). To achieve a high level of evidence, such programmes and training should be child-active, repetitive and structured with functional tasks meaningful for the child (Novak 2014). Most often, caregivers have the task of initiating the training in home settings and therefore they should be trained to understand how important it is to regularly exercise. The participants expressed an ambition to share their inter-professional knowledge with the caregivers: ‘home visits – whole team go together to the home to assist caregivers how to help the child at home’ (FG5, Statement 26). It could, for example, be *‘*not to leave the child in wheelchair the whole day [*but in*] different positions’ (FG3, Statement 8). Frequent positioning could prevent children from developing contractures and thus attend classroom activities with less pain. To enable regular training for pain management in addition to general developmental training, parent support activities such as parent evenings, support groups or newsletters had previously been offered by the schools. Among the topics discussed were the following: ‘what type of shoes to buy for the child (ankle boot rather than slipper); perceptual development of the child, and what games parent could play with child and why’ (OT, FG3, Statement 7). Enhancing caregivers’ competence in pain management could positively influence their self-worth and own feelings of being in control (Dunst & Dempsey 2007). Regrettably, the implementation of parent activities had met many obstacles, such as caregivers’ low level of education, their poor literacy skills, lack of transportation or finances to travel to the school (Adolfsson et al. 2018). As such, none of the participating schools offered courses for caregivers during the time of data collection, although it is regarded as a recommended high-level evidence intervention option. It is recommended that school-based therapists should become creative in their support to caregivers. The possibility of using mobile phones to provide parent training could be investigated, as reports indicate that 51% of South Africans own smartphones (Silver & Johnson 2018).

### Treatment strategies with lower-level evidence

All of the treatment strategies discussed so far were recommended by Novak et al. (2013). Other strategies mentioned during the focus groups could be reconsidered because of their low levels of evidence. Even though the statements were interpreted according to the context, the specific intervention was sometimes not clarified. For example, stretching could be described as child-active: ‘pain can’t be an excuse – in class, stretch, get the muscles moving, get muscles warm – the way to get children involved = moving around a lot, moving around will help with pain, stretch muscles, children are active in class, move around’ (OT, FG5, Statement 4). It could also be interpreted as a use of casting *constant stretching,* but most often it was understood as manual: ‘when she tells her leg is sore, he will pull her’ (PT, FG4, Statement 4).

Contracture prevention via manual stretching is a child-passive intervention with rather weak evidence that it can increase the range of movement, reduce spasticity or improve walking efficiency in children with spasticity (Novak et al. 2013). Passive (manual) stretching means that a child moves the targeted joint to the available end range of motion supported by a therapist or other person (Gorter et al. 2007). According to Pin, Dyke and Chan (2006), sustained stretching of longer duration would be preferable to improve range of movement and to reduce spasticity of muscles. In addition to passive stretching, three more categories can be distinguished (Gorter et al. 2007): (1) active stretching, that is, without support and preferably within daily activities, (2) therapeutic stretching with techniques based on proprioceptive neuromuscular facilitation and (3) sustained passive stretching supported by mechanical means, such as standing table or equipment such as orthoses, splinting or casting. Decisions on the most relevant stretching technique could be guided by questions (Gorter et al. 2007). If the child is able to actively move the joint to the available end range of motion, active stretching within daily activities is recommended. If not, one should focus on the child’s ability to move the joint actively. If the child lacks this ability, sustained passive stretching is recommended. If the child is able to actively move the joint, the PT can choose between, or combine, all the alternative stretching options.

Mechanisms of muscle contracture in children with CP are not clarified and evaluations of the effectiveness of different stretching techniques are unresolved, mainly because of methodological flaws in many studies, the samples studied being too small or that there are too few studies in the evaluations. Wiart et al. (2008) conclude that more research is needed to explore the structural changes that occur in the shortened muscles of children with CP and the effects of stretching practices. They recommend that PTs should consider innovative alternatives and strategies to integrate therapy with fun, everyday physical activities that the children like.

During the focus groups, therapists regularly mentioned NDT and asked for education to increase their skill levels. Neurodevelopmental therapy is a child-passive, time-consuming, widely spread motor therapy. During the past 50 years, NDT has influenced physical, occupational and speech therapies but has been evaluated as less effective in movement and functioning (Butler & Darrah 2001). Although NDT includes positioning presented as ‘reflex-inhibiting’ postures, it does not carry over into movement or function, and based on strong evidence it does not improve contracture and tone (Butler & Darrah 2001; Novak et al. 2013). Novak et al. (2013) recommend casting as a better intervention for contracture management, BoNT as more effective for tone reduction and motor learning as better for functional motor gains to help children to take control over their own movement, including balance. Therefore, and compared to NDT, it is better to identify functional, meaningful tasks to treat the children in their daily settings where they live, play and learn.

### Environmental strategies with higher-level evidence

*Casting* is an alternative and merely preventive intervention that is best used in new contractures (Novak 2014). It means that individually adapted plaster casts are applied to limbs in a stretched position, aiming to entail muscle lengthening, that is, sustained passive stretching supported by mechanical means. The fact that no statements were made about pain in upper limbs could be a proof that pain in children with CP is more often located in the legs than in arms and the discussion is limited to lower limbs.

Casting is less effective than surgery, while ‘standing frames’, which were frequently mentioned by participants as being used for a similar purpose, are even less effective than casting. ‘Position with open hips in standing frames to develop the joint’ (OT, FG3, Statement 8). However, standing frames are beneficial in the sense that they can be used by more than one child. Even though they are not individualised, standing frames could be useful for patients with low bone density or constipation (Novak et al. 2013).

## Additional resources perceived as missing

### Treatment resources

*Medication for spasticity reduction* (BoNT, baclofen, etc.): it is essential to conduct pain screening to identify pain prevalence, localisations and patterns of distribution to classify pain and consider the multiple mechanisms which may contribute to pain. However, it is also necessary that healthcare professionals immediately acknowledge pain and focus on pain reduction (Westbom et al. 2017). Botulinum toxin treatment has most often been used in 4–6-year olds to reduce spasticity (Franzen, Hägglund & Alriksson-Schmidt 2017). Healthcare professionals should offer evidence-based intervention, such as BoNT, although it is necessary to carry out intervention in a comfortable way (Nilsson et al. 2017).

The children with CP in South African schools lacked medical treatment in terms of evidence-based medicine (Nilsson et al. 2016). It was reported that it is difficult for children with CP to access effective pharmacological agents such as BoNT and baclofen, as professionals at schools are not allowed to provide children with medicine without a medical doctor’s prescription. Other challenges reported were related to, for example, financial constraints, long waiting times for medical appointments or nurses’ understanding that these pharmaceuticals caused negative side effects, such as drowsiness (Table 6). The availability of medical doctors in the school teams to better institute evidence-based care for pain management and hip surveillance programmes for children should thus be explored.

**TABLE 6:** Additional treatment and environmental resources that professionals perceived as needed to obtain and secure a satisfactory pain management for children with cerebral palsy related to the levels of the evidence of interventions for children with cerebral palsy as reported by Novak et al. (2013).

Level of evidence	Treatment resources	Environmental resources
Higher levels of evidence: S+ ‘Do it’ W+ ‘Probably do it’	Prescription of medication for spasticity reduction Accessibility to BoNT Surgery Postoperative therapy	Accessibility to external medical resources/possibility to send referrals to specialist Assistive/technical devices for standing, sitting, mobility and communication
Lower levels of evidence: W- ‘Probably don’t do it’ S- ‘Don’t do it’	NDT	

*Source*: Novak, I., McIntyre, S., Morgan, C., Campbell, L., Dark, L., Morton, N. et al., 2013, ‘A systematic review of interventions for children with cerebral palsy: State of the evidence’, *Developmental Medicine & Child Neurology* 55(10), 885–910. https://doi.org/10.1111/dmcn.12246

CP, cerebral palsy; NDT, Neurodevelopmental therapy.

*Surgery and postoperative therapy*: many children with CP may benefit from orthopaedic hip or other surgery. Single-event multilevel surgery (SEMLS) is explained by Novak et al. (2013) as:

[*A*] series of simultaneous orthopaedic procedures at different levels of the lower limb to manage contractures, optimise skeletal alignment, improve gait, and prevent ambulation deterioration or postural deterioration secondary to musculoskeletal deformities. (p. 895)

Novak et al. (2013) state that SEMLS could avoid multiple surgeries. Findings from an interview study with caregivers added that hip and scoliosis surgery reduced the children’s pain (Stähle-Öberg & FjellmanWiklund 2009). Child-active physiotherapy during at least 1 year after orthopaedic surgeries is recommended to improve functioning, for example, the child’s gait level (Table 6).

### Environmental resources

Statements concerned the availability of external medical facilities (Table 6), such as hospital care for children with CP from a young age, perceived to prevent deformities and unnecessary suffering: ‘Children were never managed and picked up from early on (primary healthcare) – deformities’ (FG1, Statement 26). Participants wanted the children to *get what they need on time*, such as faster access to surgery, postoperative care facilities and access to rehabilitation. In addition, statements involved improved hospital care for children whose caregivers did not have medical aid and giving *priority to the disabled – especially children.*

The statements suggested that – aside from orthopaedic surgeons – children in South Africa may not have sufficient access to orthotics and prosthetics services and reinforced the challenge of gaining access to external specialists (e.g. orthopaedic professionals in public hospitals) for the children’s orthopaedic needs. According to a recent report, there is no shortage of orthopaedic professionals in South Africa (Ramstrand 2018). This report stated that 793 educated prosthetics and orthotics professionals (PoPs) were available, a number that is estimated as sufficient. Compared to developing countries where the recommendation is set to 5–10 PoPs per 1 million citizens, a sufficient number for South Africa’s 56 million citizens should be 560 PoPs. As health professionals at schools for special educational needs treat the children with CP, the opportunity for them to refer the children to external resources without the long waiting times at public hospitals (as mentioned during the focus groups) should be investigated.

### Other resources perceived as missing

The participants expressed that they needed education in pain management to improve their opportunities to help the children. One such topic could be the use of evidence-based medicine.

## Action or reaction approaches

Most of the strategies in South African schools had a reaction approach, that is, strategies to intervene when a child is already experiencing pain (Adolfsson et al. 2018). In all focus groups, hip pain was mentioned as a main cause of pain, which might explain the participants’ focus on positioning. A transition to an action approach would most likely help by reducing the children’s spasticity and prevent severe contractures leading to hip displacement progressing into dislocation. Hip dislocation is preventable through early identification and intervention. As hip displacement is directly related to the level of GMFCS and most children remain at the same level from 2 years of age, this system could be used to identify hips at risk and indicate the need for interventions and systematic follow-ups in terms of hip surveillance programmes (Hägglund, Lauge-Pedersen & Wagner 2007).

Positive outcomes of hip surveillance programmes have been reported by researchers (Hägglund et al. 2007, 2014; Wynter et al. 2011). Such programmes include a standardised individual follow-up of gross motor function, clinical assessment and radiological review. It is an ongoing process that is jointly followed by PT, OT and orthopaedic surgeons and should continue until skeletal maturity so that the right interventions can be provided in a timely manner. Intervention plans include first non-surgical strategies, such as positioning, use of orthotics and assistive devices and spasticity-reducing pharmaceuticals. In addition, reconstructive hip surgery and/or SEMLS might be necessary. Preventive surgery can include adductor-psoas tenotomy, various osteotomies of the proximal femur or pelvic reconstruction (Hägglund et al. 2014). Thomason and Graham (2014) pinpoint surgery as essential, providing very good outcomes and an improved quality of life for many children with CP.

Novak (2014) refers to alternatives for effective rehabilitation intervention programmes that could also include pain management intervention. These should ‘include child-active learning-based interventions for motor and functional skill performance gains’ (Novak 2014:1151). Examples were given as bimanual therapy, CIMT, goal-directed training, home programmes and occupational therapy after BoNT. Other alternatives were orthopaedic and therapy interventions such as bisphosphonates, BoNT, casting, diazepam, fitness training and active hip surveillance, as well as compensatory and environmental interventions, such as context-focused therapy. In South African school settings, all the elements of recommended interventions are not relevant in addition to learning tasks. However, healthcare professionals who are working in the schools could keep the alternatives in mind and work for an enhanced focus on preventive pain interventions, implementation of a hip surveillance programme and improved collaboration with external doctors, such as orthopaedic surgeons.

## Limitations

The fact that the data collection was conducted 5 years ago is one of the limitations of the study. Evidence of pain management in children with CP may have changed over these past 5 years but up-to-date universal pain management strategies have been described and discussed in the article. Nonetheless, this study clearly showed a discrepancy between published guidelines and clinical practice within school settings. As a more recent meta-review has not been found, the scientific knowledge is deemed valuable even today. Another possible change during the years concerns the intervention options in the schools. Therefore, a follow-up of this study with new data collection evaluating whether the conditions may have changed since data collection is proposed. As the study was limited to the Gauteng province of South Africa, the results cannot be generalised for the whole country.

## Conclusion

This study showed a discrepancy between published evidence and clinical practice for the management of chronic nociceptive musculoskeletal pain in children with CP as reported by professionals working in South African school settings. The results showed that even if evidence of best practice in pain management exists, it may not guarantee that children with CP receive this management in their daily care within school settings. If chronic nociceptive musculoskeletal pain is not acknowledged and treated, it might affect the children’s learning and development.

### Recommendations for implementation in practice

Improved knowledge about and accessibility to pain management interventions are needed, such as:

- education about evidence-based practice for interventions of children with CP
- systematic follow-ups of the health status of children with CP, focusing on the hip displacement and muscle contractures
- orthopaedic resources such as surgeons, prosthetics/orthotics professionals and individually adapted orthotic devices
- shorter waiting times for doctors’ appointments
- medication for spasticity reduction, for example, BoNT and baclofen
- better opportunities for collaboration with caregivers
- treatment integrated in daily settings where children live, play and learn.

## Appendix 1

**TABLE 1-A1:** Levels of evidence (GRADE†) for intervention options relevant for pain management. Descriptions from Novak et al. (2013:888–897) or Novak (2014:1148–1151).

Intervention	Health and secondary prevention approach	Compensatory and environmental approach
**S+. Strong evidence for (‘Do it’)**		
Spasticity management	Botulinum toxin (BoNT)	A drug injected into overactive spastic muscles to block local spasticity. The drug is also used to manage local dystonia.
	Diazepam	An oral medication used for managing global spasticity.
	Selective dorsal rhizotomy	A neurosurgical procedure used to selectively sever nerve roots in the spinal cord, to relieve spasticity. The procedure is only effective for children with pure spastic diplegia and good pre-surgical muscle strength and control. The approach can worsen ambulation in children not meeting these strict inclusion criteria.
Contracture management	Hip surveillance (maintaining hip joint integrity)	Active hip surveillance and treatment for hip joint integrity to prevent hip dislocation. Can include a combination of orthopaedic surgery, botulinum toxin, selective dorsal rhizotomy and physical therapy. Management and oversight of the hips by an orthopaedic surgeon is recommended.
	Casting – lower limb	Plaster casts are applied to limbs in a stretched position to induce muscle lengthening. The amount of lengthening possible is substantially less than in a surgical approach and is best used in new contractures.
Motor activities and function	Constraint-induced movement therapy (CIMT)	Child-active, repetitive structure training in the use of the hemiplegic upper limb by constraining the dominant hand. The approach is equally effective as bimanual training. A dose of 3060 h of therapy within a 6–8-week period is needed to be effective. Both approaches are equally effective.
	Bimanual training	Child-active, repetitive and structured practice in walking, gross motor tasks (e.g. bike riding) or self-care tasks (e.g. dressing) designed to meet a goal meaningful for the child. In goal-directed training, the tasks and the environment are also changed to promote skill acquisition. It can be delivered via a home programme.
	Goal-directed functional training	Therapeutic practice of goal-based tasks by the child, led by the parent and supported by the therapist, in the home environment.
	Home programmes	Parent (or caregiver) training is included – that is, educating and coaching caregivers to change their child’s behaviour or skills, plus improve parenting.
	Context-focused therapy	The task or the environment is changed (but not the child) to promote successful task performance.
	Occupational training following BoNT	Involves child-active practice of hand function and functional tasks (chosen by the child as important) after BoNT to reduce muscle spasticity that augments the effect of BoNT alone.
	Pressure care	Prevention of pressure ulcers via good positioning, repositioning and provision of suitable support surfaces.
**W+. Conditional for (‘Probably do it’)**		
Spasticity management	Tizanidine oral	Antispasticity medication.
	Intrathecal baclofen (ITB)	Used to manage global severe spasticity and dystonia. Baclofen is delivered directly to the spine (and central nervous system) via a pump surgically implanted within the abdomen.
	Baclofen oral	An oral medication used to manage global spasticity and dystonia. In the oral format, the doses need to be high to induce a clinical effect, but this has to be balanced against the side effect of drowsiness.
Contracture management	Ankle foot orthotics (AFOs)	A removable external device is worn over the ankle and foot designed to prevent or manage ankle contractures as well as promote gait stride length in ambulant children.
	Orthopaedics (hip and other surgery)	Orthopaedic surgery involves surgical prevention or correction of musculoskeletal deformities, for example, muscle lengthening, osteotomies.
	Orthotic hand	Immobilisation hand splinting is a health and secondary prevention approach that uses custom-moulded thermoplastic or neoprene hand orthotics designed to hold the hand in a position of stretch to prevent or manage contractures.
	Single-event multilevel surgery (SEMLS)	A specific orthopaedic surgery where a series of simultaneous orthopaedic procedures at different levels of the lower limb to manage contractures, optimise skeletal alignment, improve gait and prevent ambulation deterioration or postural deterioration secondary to musculoskeletal deformities are performed. The advantage of this surgical approach is that multiple surgeries are avoided and outcomes are superior.
	Hand surgery	Involves surgical prevention or correction of musculoskeletal deformities, for example, muscle lengthening and tendon transfer. Improve hand function and alignment.
Motor activities and function	Early intervention (EI)	Very variable. Contemporaneous EI is a child-active, repetitive and structured practice of gross motor, hand function and learning tasks.
		Traditional early intervention involved general early learning stimulation or child-passive interventions where the therapist passively facilitated normalised movement patterns with the aim of inducing an upstream benefit to functional activities – traditional early intervention approaches are no longer recommended based on current neuroscience evidence.
	SEMLS and therapy	A series of simultaneous orthopaedic procedures to optimise skeletal alignment and prevent ambulation deterioration secondary to musculoskeletal deformities. Child-active physical therapy is recommended for the first year after surgery to enable children to initially return to their pre-surgical gait level and surpass their pre-surgical gait level.
	Biofeedback	Biofeedback is electronic feedback about muscle activity to teach voluntary muscle control and is therefore a child-active approach.
	Hydrotherapy	Therapeutic activities in heated water, where the water provides weightlessness for ease of movement but also resistance for muscle strengthening.
	Electrical stimulation (ES, NMES, FES)	Electrical stimulation of a muscle through a skin electrode to induce passive muscle contractions for strengthening or motor activation.
	Hippotherapy	Therapeutic horseback riding. It is assumed that the horse’s movement simulates and automatically transfers to the pelvic tilt required during walking. For non-ambulant children, sometimes the goal of hippotherapy is to promote postural control for supported sitting.
	Assistive technology	Equipment or devices to improve independence, for example, in activities of daily living or participation in education.
	Seating and positioning	Assistive technology that enables a person to sit upright with functional, symmetrical or comfortable posture to enable function.
**W-. Conditional against (‘Probably don’t do it’)**		
Spasticity management	Dantrolene	Antispasticity medication
	Intramuscular injections of alcohol or phenol	Muscular injections to induce chemical denervation for treating local spasticity
	Casting	Plaster casts applied to limbs to reduce spasticity
Contracture management	Stretching manual	Use of an external passive force (e.g. parent) exerted upon the limb to move it into a new and lengthened position
Motor activities and function	Conductive education (CE)	A Hungarian educational classroom-based approach to teaching movement using rhythmic intention, routines and groups
	Vojta	Therapist-applied pressure to defined zones on the body while positioned in prone, supine or side lying, where the stimulus leads to automatically and involuntarily complex movement
	TheraSuits	Used for elimination of pathological reflexes and establishing new, correct and functional patterns of movements (Author’s comment)
**S-. Strong evidence again St (‘Don’t do it’)**		
Spasticity management	Hip bracing	Includes a variety of hip stabilisers and hip joint supports (Author’s comment).
Contracture management	Neurodevelopmental therapy (NDT, Bobath)	Direct, passive handling and guidance to optimise function
Motor activities and function	Neurodevelopmental therapy (NDT, Bobath)	Direct, passive handling and guidance to optimise function
	Sensory integration (SI)	Therapeutic activities to organise sensation from the body and environment to facilitate adaptive responses (e.g. hammock swinging)
	Hyperbaric oxygen (HBO)	Inhaled 100% oxygen inside a pressurised hyperbaric chamber

*Source*: Novak, I., McIntyre, S., Morgan, C., Campbell, L., Dark, L., Morton, N. et al., 2013, ‘A systematic review of interventions for children with cerebral palsy: State of the evidence’, *Developmental Medicine & Child Neurology* 55(10), 888–897. https://doi.org/10.1111/dmcn.12246; Novak, I., 2014, ‘Evidence-based diagnosis, health care, and rehabilitation for children with cerebral palsy’, *Journal of Child Neurology* 29(8), 1148–1151. https://doi.org/10.1177/0883073814535503

FES, functional electrical stimulation therapy; NMES, neuromuscular electrical stimulation.

† , Grading of Recommendations Assessment, Development, and Evaluation (GRADE) is developed to assess efficiency and formulate recommendations along a four-part continuum.
